# Supplementary figures and images for: How social learning shapes the efficacy of preventative health behaviors in an outbreak
Source: PLoS One. 2022 Jan 11;17(1):e0262505. doi: 10.1371/journal.pone.0262505 (PMC8752029; doi:10.1371/journal.pone.0262505)

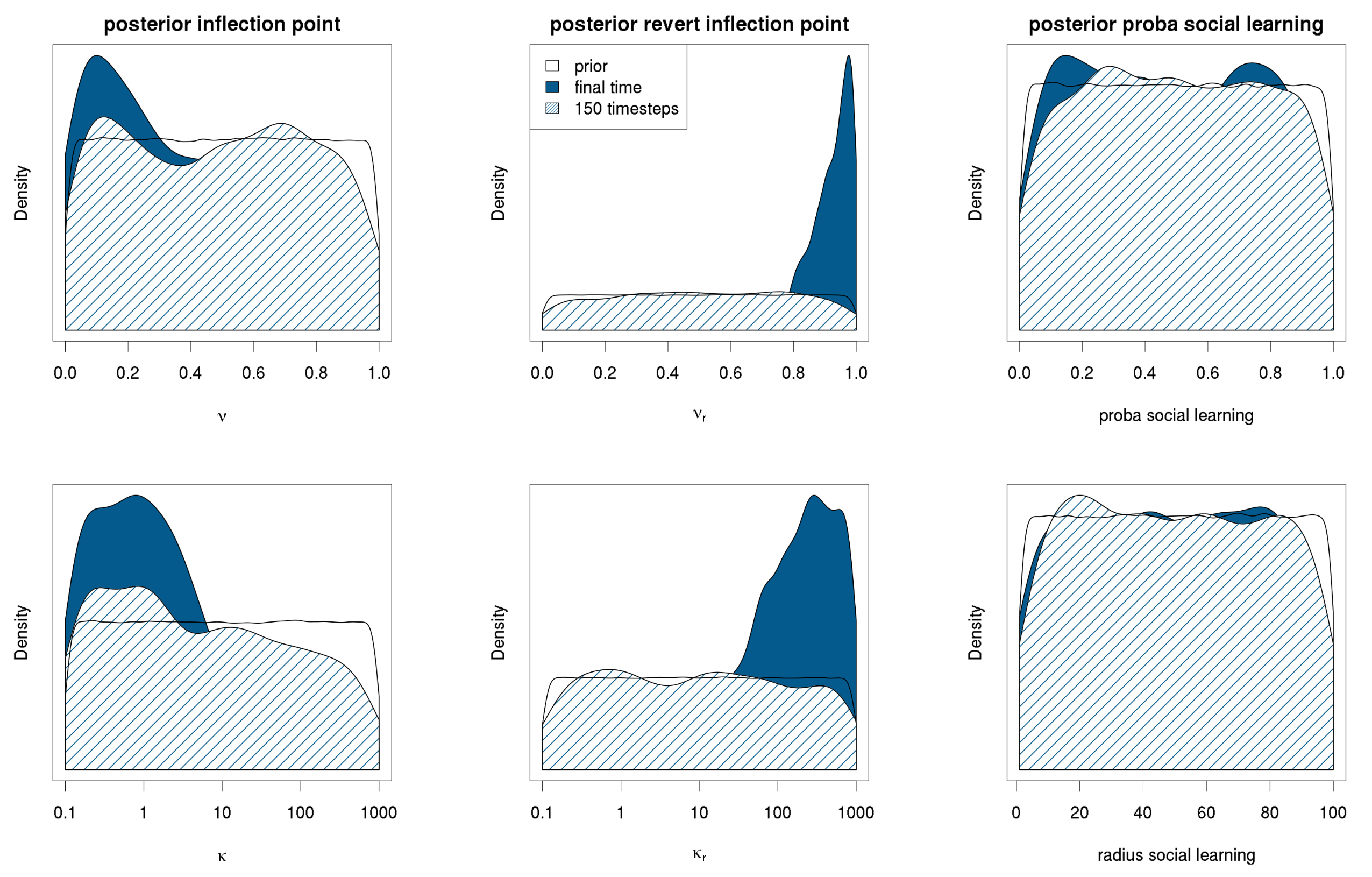

Supplement: S1 Fig — All posterior distributions for the 6 parameters used in our simulations. The white area represents the distribution of the parameters of all simulations (the priors), the blue area represents the distribution for the top 1000 simulations rank using δ while the shaded area represent the parameters of the 1000 simulations with the lowest number of infected people after 150 timesteps.Left column represents the parameters value to switch from Non-Adherent to Adherent, middle column from Adherent to Non-Adherent, and right column the paramaters that defines the probability and the radius of social learning. (TIFF) [file pone.0262505.s001.tiff]

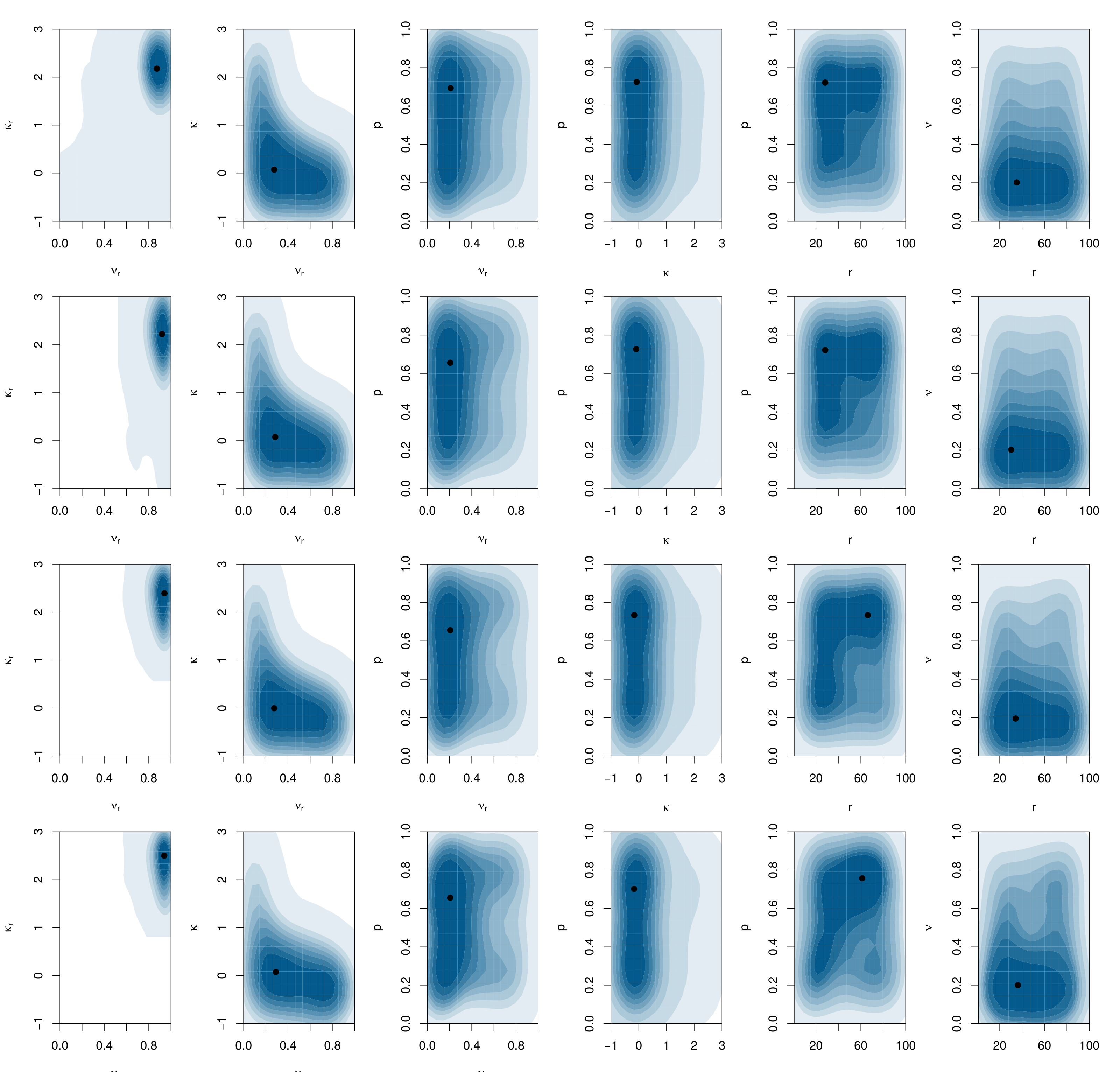

Supplement: S2 Fig — Joint posteriors of representative pair of parameters of the model for different level of what we consider as the “best” simulations. Each distribution represent the parameter distribution of simulations for which the metric δ is ranked below different level, from top to bottom: 10, 000;5, 000;2, 500;1, 000. The 2d areas represent the High Density Regions, ie the smallest regions within which falls a certain percentage of the distribution of the parameters our selected simulation. The lighter areas represent the area within which all the simulations are distributed and darker areas represent regions for smaller HDR. (TIFF) [file pone.0262505.s002.tiff]
