# Supplementary material for: How social learning shapes the efficacy of preventative health behaviors in an outbreak
Source: PLoS One. 2022 Jan 11;17(1):e0262505. doi: 10.1371/journal.pone.0262505 (PMC8752029; doi:10.1371/journal.pone.0262505)
Supplement: S1 Text — This text describes and illustrates results for 4 variants of the original model. (PDF) [file pone.0262505.s004.pdf]

# Different Model of Social Learning and Initial distribution

Supporting Text S1 for *"How Social Learning Shapes Preventative Health Behaviors in an Outbreak"*

S. Carrignon, R. A. Bentley, M. J. Silk, N. H. Fefferman

Aside the simulations run with the setup described in the main paper we run sets of slight variation where:

- Instead of allowing each individual to copy only healthy individuals of the same age (Best SLS), the social learning strategy allows them to copy the behaviour of any individuals of the same age (Random SLS),
- The initial distributions of behaviors varies (0% Adherent - the normal setup, 15% Adherent and 100% Adherent),
- Learning (individual and social learning) starts after 100 time steps.
- Social learning is less efficient (higher probability of infection of Adherents individuals $P_a$).

We did not try all combinations of all variations, the table 1 summarizes which ones are discussed in the following sections. The variant A is the one presented in the main paper. The numbers of simulations ran for each variants are as follows:

- Variant A : $826,892$ simulations
- Variant B : $158,400$ simulations
- Variant C : $251,000$ simulations
- Variant D : $130,199$ simulations
- Variant E : $153,499$ simulations
- Variant F : $309,998$ simulations
- Variant G : $99,998$ simulations
- Variant H : $99,998$ simulations

Figure 1 shows the distributions of $\delta$ , $\tau$ and $I_{max}$ for all simulations of all model's variants. As we can see all variants generate generally similar outputs, except for the variants F and H (and G at a lower level). In F, learning (social and observational) is impossible until the time step 100, thus the mode of $\tau$, the time to reach the maximum number of infected people, is slightly shifted by around 40 time steps. In this variant all individuals are adherent to social distancing and stick to it for the first 100 time steps, which slows down the infection. Nonetheless, as soon as we relax and bring back the learning abilities,

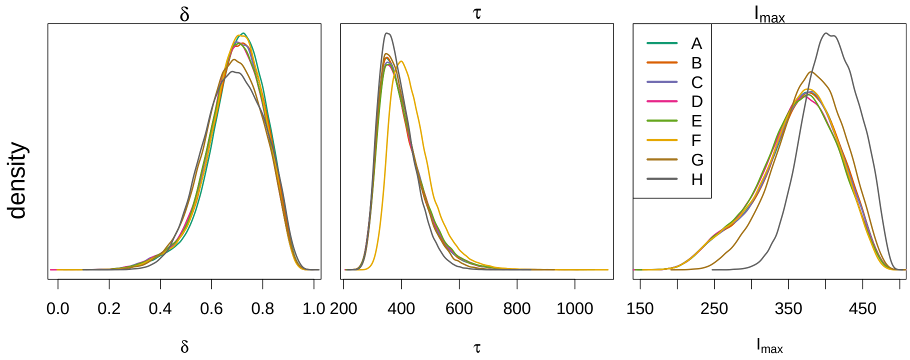


Figure 1: Distribution of the metrics described in the section Results for all simulations for all model (approx 100k simulations per model).

the exact same properties are observed than for the other simulations: we will reach the same $I_{maxx}$ and $\delta$, and the combination of parameters that allow us to reach such results are the same than for the over variants, as the posteriors shown in the next sections illustrate. Regarding H and as described in the main text of the paper, social distancing being less effective, the maximum number of infected people ($I_{max}$) is higher, which is also true for G but with a lower difference. On the other hand, the posteriors distributions for the parameters that dictates social learning remains the same, though the distributions are broader than for the over models, as social distancing becomes less and less useful to flatten the curve, and thus the behavior of learners matters less and less.

### Results for all variants

Figures 2-9 show the marginal posteriors for all parameters in each model variant. Figures 10-17 show the joint posteriors of the parameters used for observational learning in each model variant. All figures from 2 to 17 confirm that the overall results are not affected by the Social Learning Strategies nor the initial distribution of behaviors, the selected posteriors are approximately the same for all the variants tested.

| | | % Adherents at t=0 | | | | $P_a$ |
|---|---|---|---|---|---|---|
| SLS | learning start | 0 | 15 | 50 | 100 | 0.1 |
| Healthy | 0 | A | B | | C | 0.1 |
| Random | 0 | D | E | | | 0.1 |
| Healthy | 100 | | | F | | 0.1 |
| Healthy | 0 | G | | | | 0.2 |
| Healthy | 0 | H | | | | 0.6 |

Table 1: Summarize all variants of the models

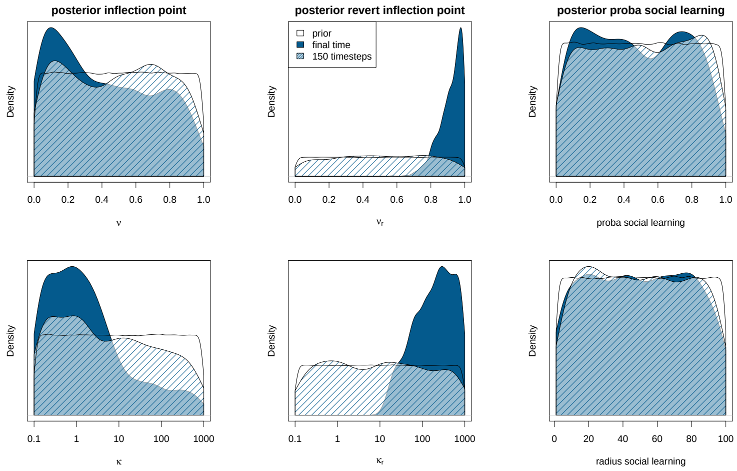


Figure 2: Same as figure 6, variant A

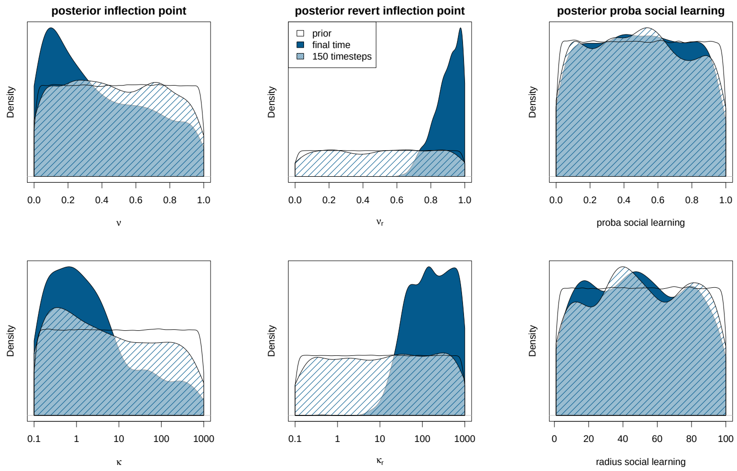


Figure 3: Same as figure 6, variant B

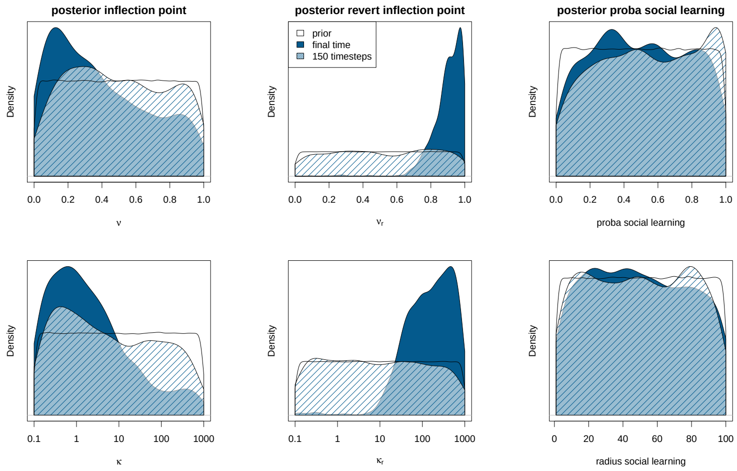


Figure 4: Same as figure 6, variant C

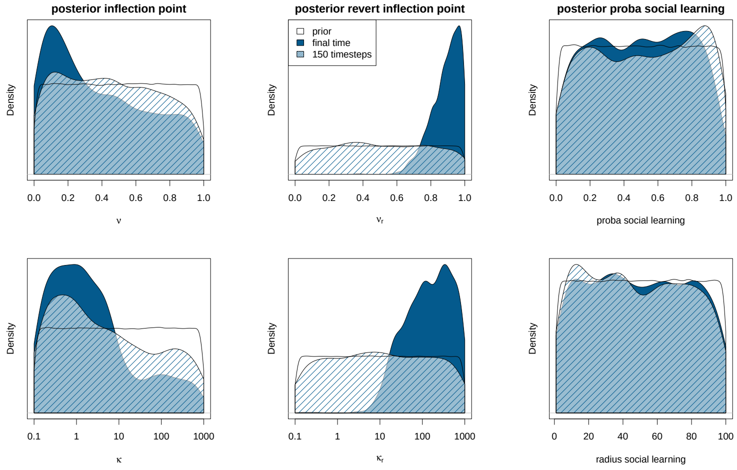


Figure 5: Same as figure 6, variant D

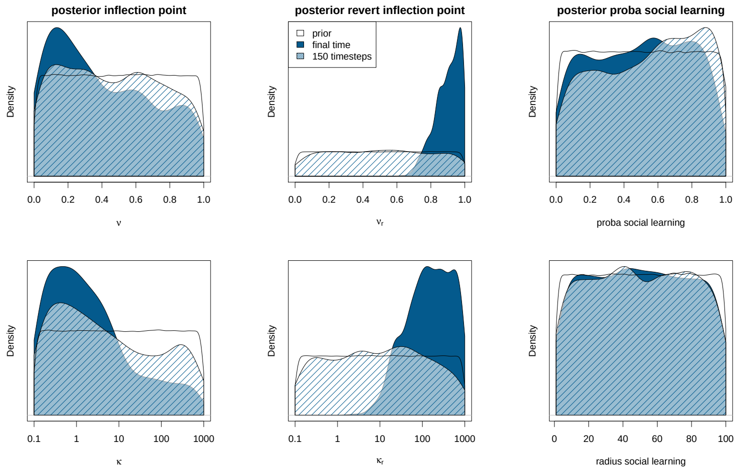


Figure 6: Same as figure 6, variant E

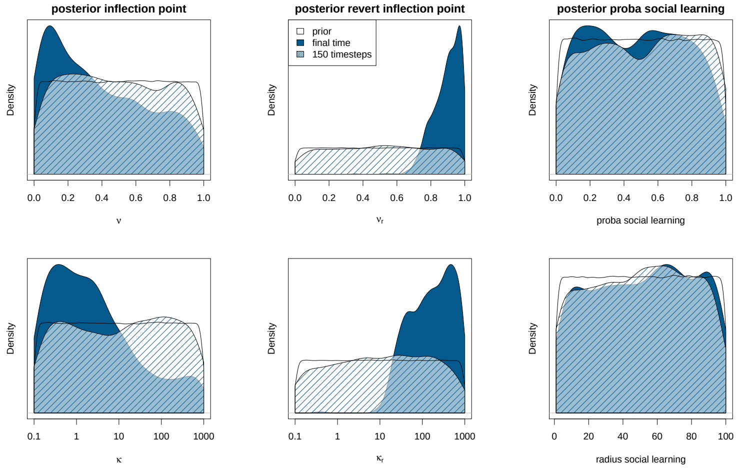


Figure 7: Same as figure 6, variant F

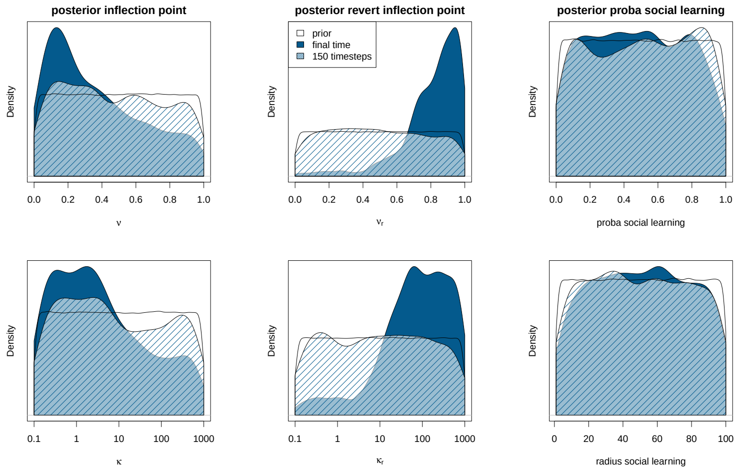


Figure 8: Same as figure 6, variant G

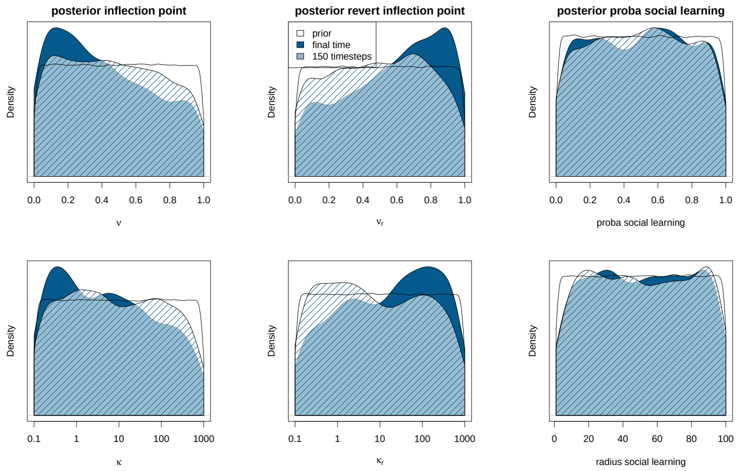


Figure 9: Same as figure 6, variant H

Parameters to switch from Non-Adherent to Adherent | Parameters to switch from Adherent to Non-Adherent

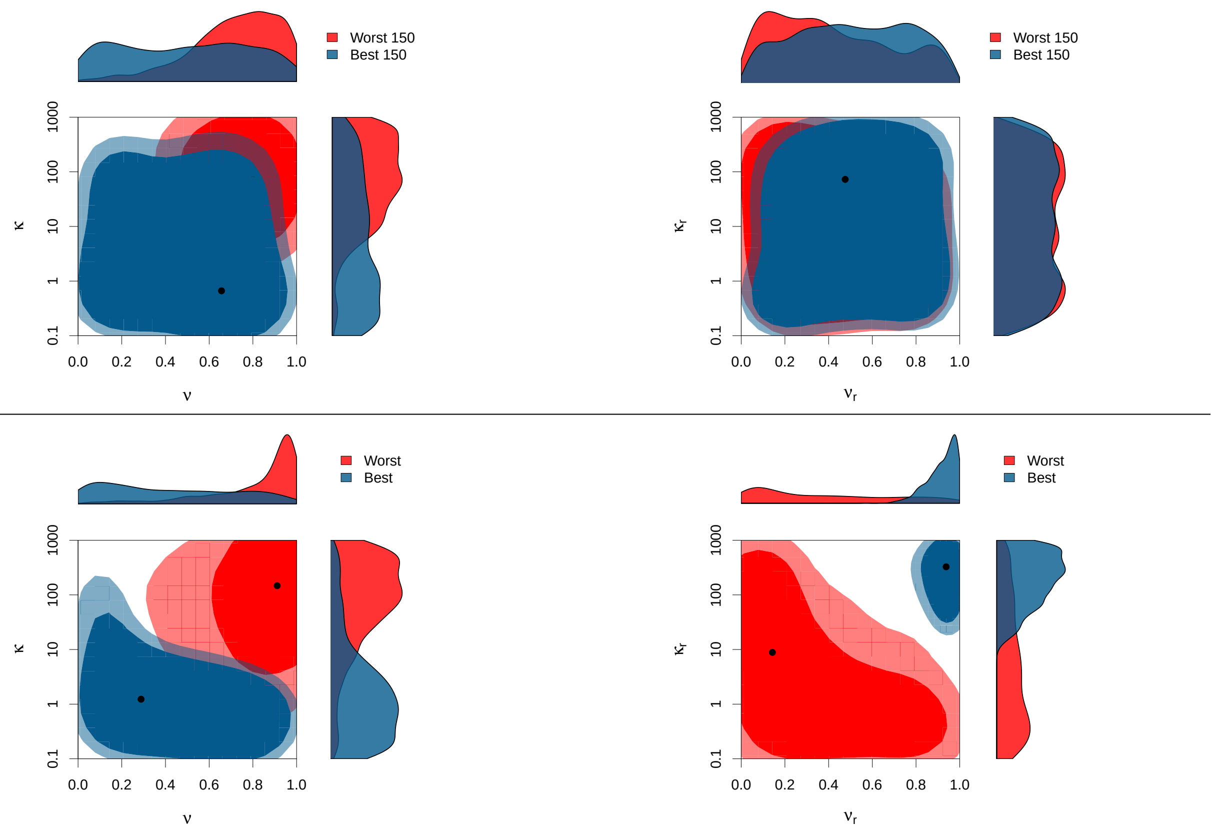


150

1500

Figure 10: Variant A

<table>
<tr><th></th><th>Parameters to switch from Non-Adherent to Adherent</th><th>Parameters to switch from Adherent to Non-Adherent</th></tr>
<tr><td>150</td><td>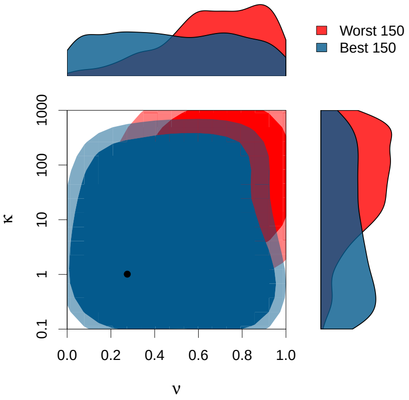
</td><td>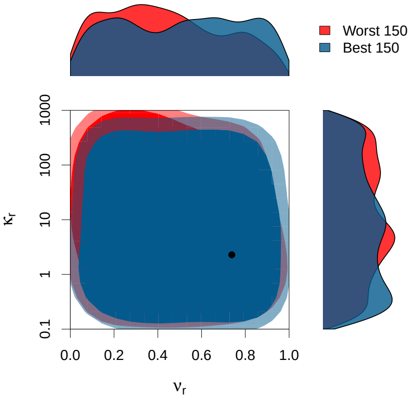
</td></tr>
<tr><td>1500</td><td>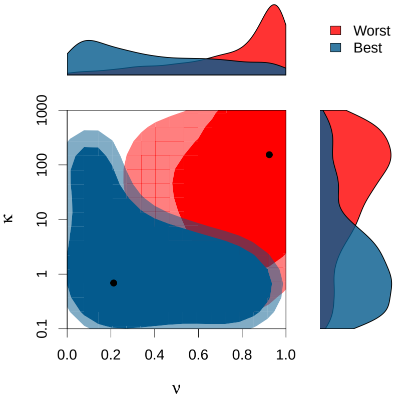
</td><td>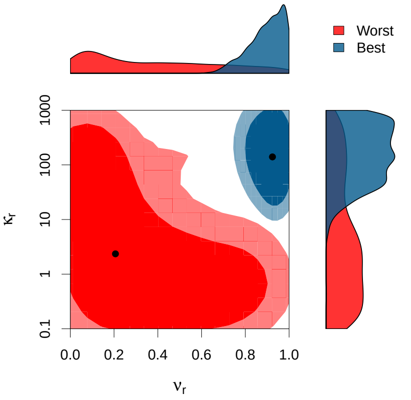
</td></tr>
</table>

Figure 11: Variant B

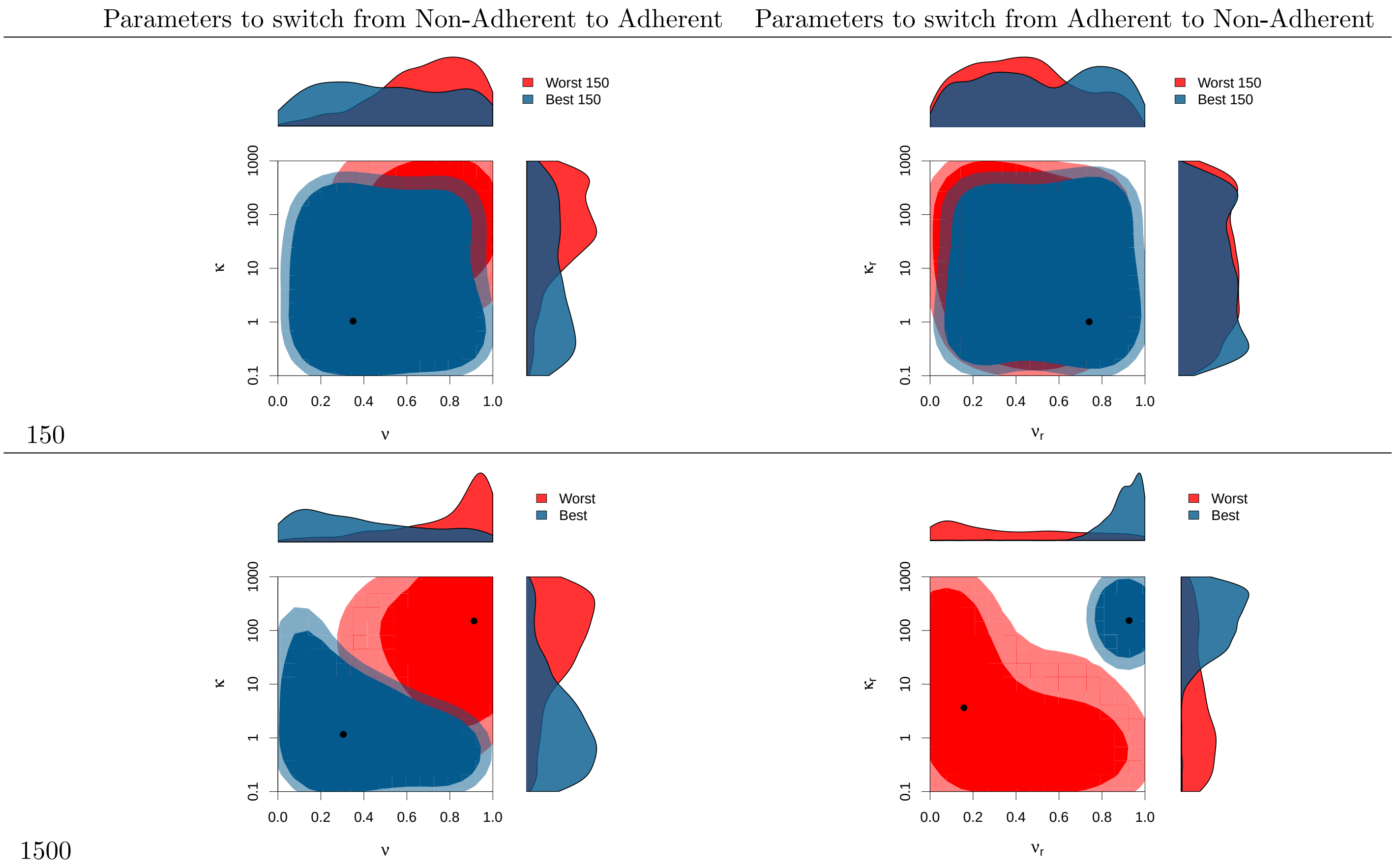


Figure 12: Variant C

| | Parameters to switch from Non-Adherent to Adherent | Parameters to switch from Adherent to Non-Adherent |
|---|---|---|
| 150 | 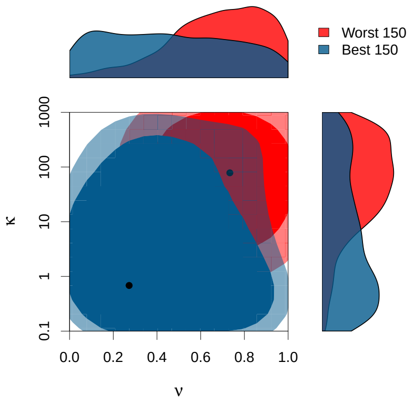 | 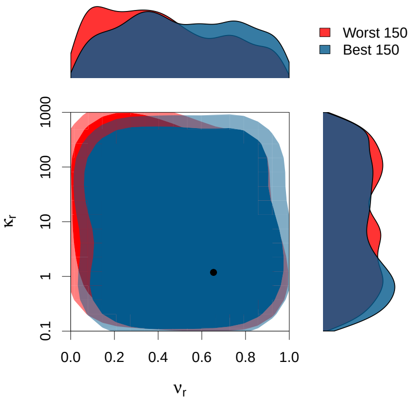 |
| 1500 | 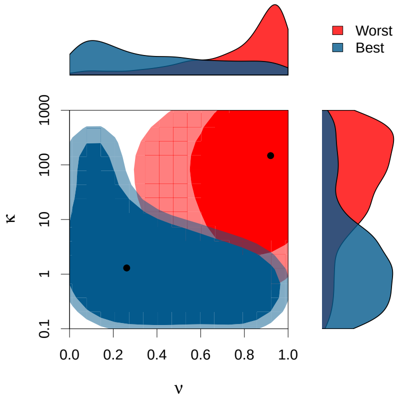 | 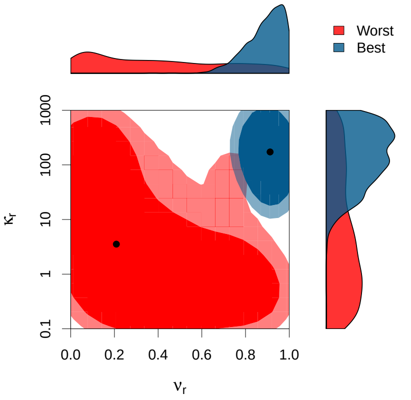 |



Figure 13: Variant D

| | Parameters to switch from Non-Adherent to Adherent | Parameters to switch from Adherent to Non-Adherent |
|---|---|---|
| 150 | | |
| 1500 | | |

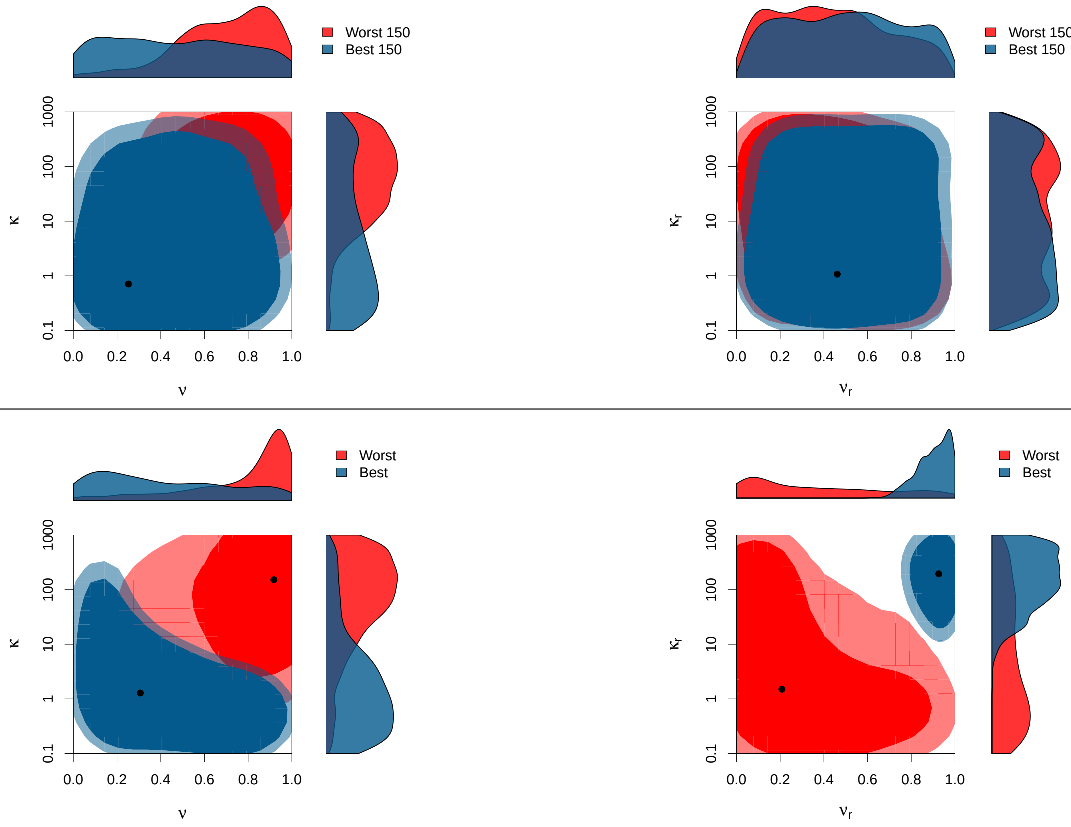


Figure 14: Variant E

| | Parameters to switch from Non-Adherent to Adherent | Parameters to switch from Adherent to Non-Adherent |
|---|---|---|
| 150 | 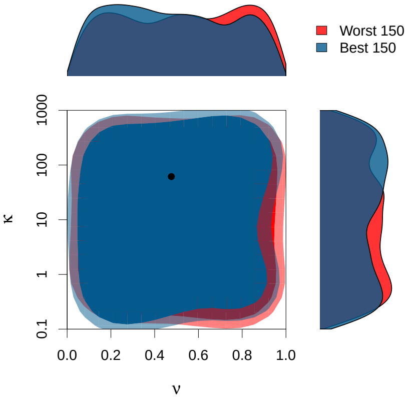 | 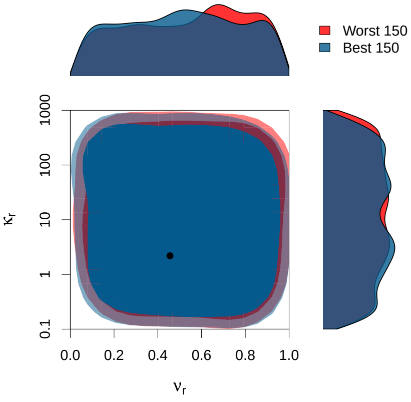 |
| 1500 | 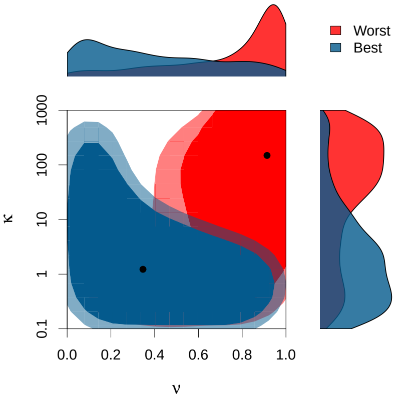 | 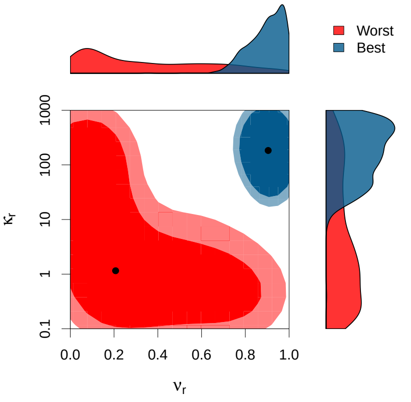 |

Figure 15: Variant F

| | Parameters to switch from Non-Adherent to Adherent | Parameters to switch from Adherent to Non-Adherent |
|---|---|---|
| 150 | 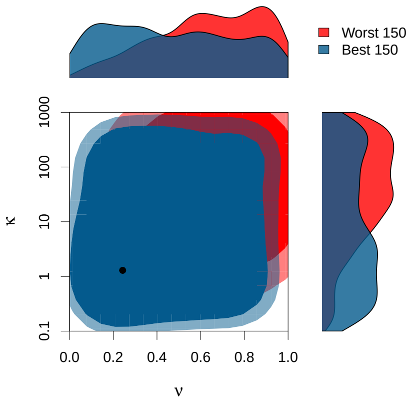 | 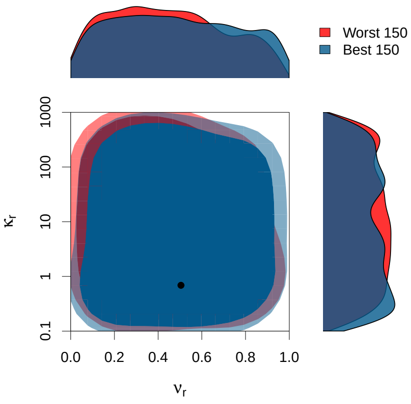 |
| 1500 | 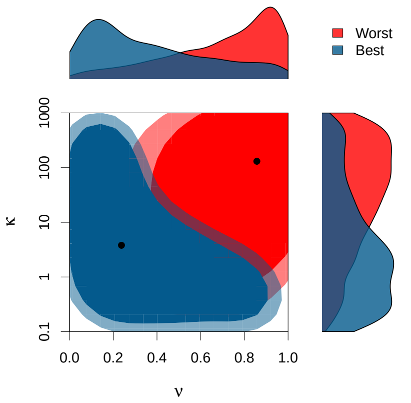 | 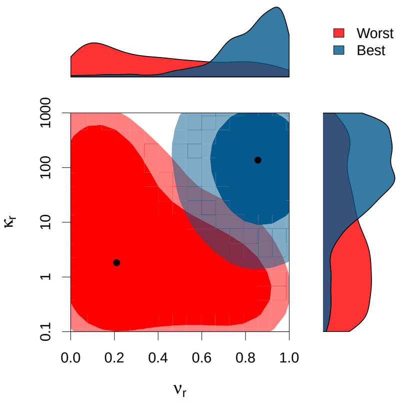 |









Figure 16: Variant G

Parameters to switch from Non-Adherent to Adherent | Parameters to switch from Adherent to Non-Adherent

150

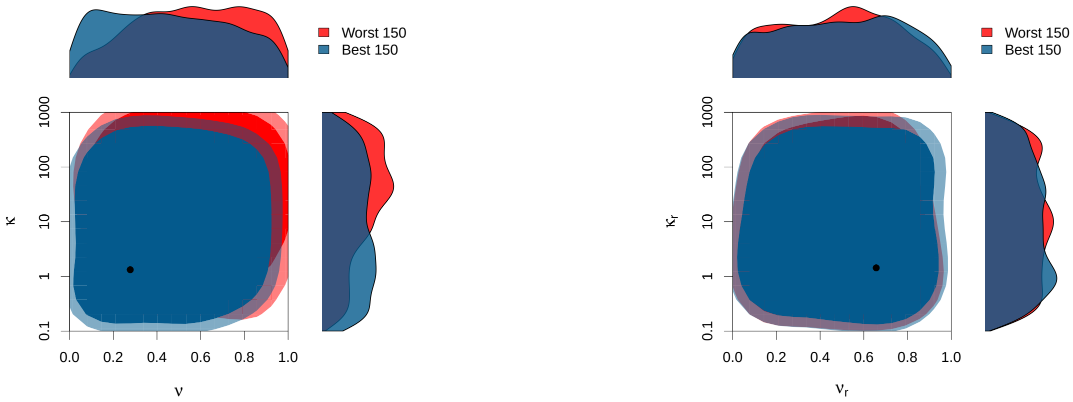


1500

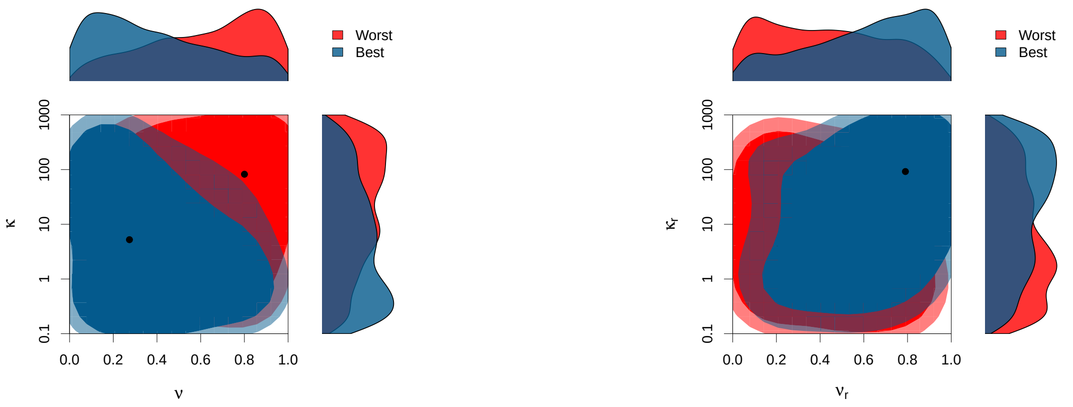


Figure 17: Variant H
