# Supplementary material for: How social learning shapes the efficacy of preventative health behaviors in an outbreak
Source: PLoS One. 2022 Jan 11;17(1):e0262505. doi: 10.1371/journal.pone.0262505 (PMC8752029; doi:10.1371/journal.pone.0262505)
Supplement: Attachment — Submitted filename: Response_to_reviewers.pdf [file pone.0262505.s005.pdf]

Manuscript Number: PONE-D-21-16649
"How social learning shapes the efficacy of preventative health behaviors in an outbreak"

Reviewer's comments are in red, our responses in black, and changes to manuscript in blue.

Reviewer #1:

1. The replication package seems to be missing files required for all of the included scripts to run. I could not find abmEpi.R and covid.masks_small.csv in the linked github repository and I could not find S1 File in the list of supplements I got. I could only get the Intro vignette to run and was not able to get the other vignettes used for replicating the graphs to run. I was able to get slsirSimu() and generatePopulation() to work as expected. The visualizations provided by the visu flag were well designed and informative.

A new vignette complete has been added to the repository and a compiled version can be accessed through https://simoncarrignon.github.io/slsir/vignettes/paperPLOSONE.html. This should allow anyone to reproduce all figures and analysis of the paper.

2. Having the behavioral state impact how effectively infected individuals spread the pathogen could help the model better generalize to the COVID-19 pandemic. While there are some behavioral interventions that prevent infection in the person doing them (self isolation), many of these behaviors also make it less likely for the infected to spread the disease (mask wearing). My hunch is that this would increase the likelihood of observing the Icarus Paradox.

Agreed. These kinds of behavioral variations are part of ongoing work, where we try to build on the simple foundation of the model in this paper.

I would be interested to see how sensitive the results are to the parameter P_a. If P_a were higher, then more infections would "breakthrough" the social distancing and there would be more infections even with high levels of social distancing adoption. This could impact how the Icarus effect manifests. It could strengthen the results to show how social learning impacts behavioral choice, when the behaviors have different levels of efficacy in reducing spread.

We address this with tests (see below), but note that it effectively nullifies our research question on the effect of heterogeneous social distancing on the spread of a pandemic. Increasing P_a reduces the efficiency of social distancing, and hence the phenomenon we intended to measure disappears. To illustrate it we have simulated two additional model variants, labelled H and G (supplementary material), where P_a is respectively 0.2 and 0.6. The posterior distributions of the switching parameters illustrates well how the adoption mechanism become less important as the curves become more similar to the priors (Figure 8 and 9 in S1_text.pdf), whereas the overall number of infected individual increases (Fig1 in S1 text, curves G and H), tending toward cases where flattening the curve through social learning becomes impossible.

Minor Comments: 1. The slsirSimu() and generatePopulation() are private which can make the less user friendly for running your own simulations with different parameters 2. There are some typos in the documentation for the functions and the package metadata is still the default package metadata. 3. It would be helpful to clarify which scripts / markdown documents are essential for replicating the results in the paper (especially the scripts in the exec folder).

Regarding all three minor points: The package has been updated. The main functions are now exported and public. The documentation has been clarified. Scripts in the exec/ folder have been translated as vignettes with clearer explanations.

Reviewer #2:

However, my major concern is the lack of plateaus and secondary waves in the simulations as is observed in real epidemics. In fact, many recent papers have observed that it is precisely the behavioral factors and joint evolution of human response and epidemics that lead to emergence of second waves and plateaus. …. [1] Weitz, Joshua S., et al. "Awareness-driven behavior changes can shift the shape of epidemics away from peaks and toward plateaus, shoulders, and oscillations." Proceedings of the National Academy of Sciences 117.51 (2020): 32764-32771. [2] Toxvaerd, Flavio MO. "Equilibrium social distancing." University of Cambridge, Working Paper (2020). [3] McAdams, David. "The Blossoming of Economic Epidemiology." Annual Review of Economics 13 (2021). [4] Pedro, Sansao A., et al. "Conditions for a second wave of COVID-19 due to interactions between disease dynamics and social processes." Frontiers in Physics 8 (2020): 428. It will be important to explore why the proposed model does not capture these important phenomena. If it can be shown that social learning or lack of transparency that led to early containment of first wave, but immediate reverting to non-adherence led to emergence of second waves, then the proposed model would be better justified and the paper would be stronger.

These are excellent studies, all of which we now cite in context (see below). Clearly, progress is being made on multiple fronts, and our study constitutes one among several different approaches. Regarding a second wave, this can be modelled via a step-function representing a system-wide change in social distancing (e.g. lockdowns or school closures), whereas our individual-based model does not have this mechanism. Hence this phenomenon would require additional parameters and a full re-implementation of the model, which is outside the scope of current work (although we would be interested to apply in future). In response to these important points, we have added the following paragraphs to our Discussion:

> Here we have focused on public health measures that could be quickly adopted, in the initial case where sweeping lockdowns are not politically feasible. Our model examines the initial transient coupled dynamics directly following the introduction/emergence of a novel pathogen threat. The model proposed here focuses exclusively on the first, major wave of infections. While our contribution has been to couple the social and individual responses, we note that even in an asocial awareness model, a pandemic peak can become a prolonged plateau rather than a sharp peak, if individual transmission decreases in response to awareness of the disease at a population level (Funk et al. 2010; Weitz et al. 2020; Toxvaerd 2020).
>
> The past two years have witnessed multiple surges of COVID-19 in many countries, including the U.S., for a diverse range of socio-political reasons (McAdams 2021). Our base model presented here— focused on a single coherent, population-level response— would not, on its own, explain the observed secondary waves of COVID-19. In 2020-2021, different municipalities quickly diverged in their response to COVID-19 risks; lockdowns lasted different durations of time (Weitz et al. 2020), political discourse became divisive (McAdams 2021), and later new strains such as Delta altered the disease epidemiology. In a mathematical model, one way to induce a second wave is to introduce a step function, representative of a centralized decision to remove lockdowns (i.e. increase spread) when public support for closure drops below a certain threshold. Pedro et al. (2020) introduce such a model, focused on centralized decisions such as school closures, that also includes social learning dynamics underlying these centralized “on-off” decisions. Since our model is based on individual, rather than centralized, decisions, this step-change instigating a second wave would not occur in our base model without more complex modifications, although this would be a valuable topic for future research. In particular, we anticipate future work where studies build on our work to investigate the various reactions that could occur as outbreaks progress. Future exploration of the model could integrate these aspects, by adding asymptomatic individuals, the apparition of new variants, more complex population structure and the possibility for recovered individuals to be infected again after a certain time. We could then explore which social learning features can mitigate subsequent waves.
